# Molecular docking and pharmacological investigations of folenolide for its analgesic, anti-inflammatory, and antipyretic applications

**DOI:** 10.3389/fphar.2025.1658610

**Published:** 2025-11-19

**Authors:** Faryal Liaqat Ali, Fazli Khuda, Atif Ali Khan Khalil, Asif Jan, Muhammad Asif Khan, Ahmed Nadeem, Abdur Rasheed, Syed Shaukat Ali, Asmat Ullah

**Affiliations:** 1 Department of Pharmacy, University of Peshawar, Peshawar, Pakistan; 2 Department of Biotechnology, Yeungnam University Gyeongsan, Gyeongsan-si, Republic of Korea; 3 Saidu Group of Teaching Hospital Saidu Sharif Swat, Peshawar, Pakistan; 4 Department of Pharmacy, Sarhad University of Science and IT, Peshawar, Pakistan; 5 Department of Pharmacology and Toxicology, College of Pharmacy, King Saud University, Riyadh, Saudi Arabia; 6 Spinghar Medical University Jalalabad, Jalalabad, Afghanistan

**Keywords:** folenolide, acute toxicity, inflammation, analgesic, antipyretic, molecular docking

## Abstract

**Background:**

Folenolide, a novel compound, was investigated for its pharmacological potential, focusing on its analgesic, anti-inflammatory, and antipyretic effects, along with its safety profile and potential molecular targets, using *in vivo models* and molecular docking studies.

**Methods:**

*In vivo* mouse models were used to assess acute toxicity and analgesic, anti-inflammatory, and antipyretic activities. Molecular docking simulations were conducted against key targets, including COX-2, lipoxygenase, opioid, histamine, and tankyrase receptors, to explore their potential mechanisms of action.

**Results:**

Folenolide showed no signs of acute toxicity. The analgesic model exhibited significant peripheral analgesia in the acetic acid-induced writhing test (31.07% and 50.13% inhibition at 3 and 6 mg/kg, respectively; *p* < 0.01) and potent central analgesia in the hot-plate test (maximum effect at 6 mg/kg; *p* < 0.0001). Formalin test results confirmed both neurogenic and inflammatory phase inhibition (69.17%–78.26%). Anti-inflammatory efficacy was marked in carrageenan-induced paw edema (84.3%–94.98% reduction), histamine-induced edema (87.29%–88.6%), and xylene-induced ear edema (6.19%–8.19% weight gain suppression) (*p* < 0.05). Folenolide also displayed antipyretic effects, significantly reducing the rectal temperature in yeast-induced fever models. Molecular docking revealed favorable binding affinities (ΔG ranging from −4.4 to −5.9 kJ/mol) with key targets.

**Conclusion:**

Folenolide exhibits strong analgesic, anti-inflammatory, and antipyretic activities with a favorable safety profile. Its pharmacodynamic effects are supported by its molecular interactions with cyclooxygenase (COX), opioids, and histamine receptor targets. These findings highlight its potential as a promising therapeutic agent for pain, inflammation, and fever management.

## Graphical Abstract

**Figure:**
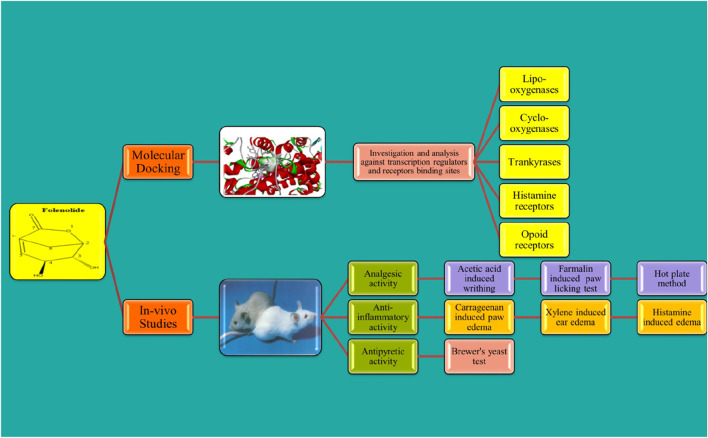

## 1 Introduction

Historically, natural products have been the main source of medicine used to treat various human ailments. In addition to offering a number of innovative and potent chemical entities for use in modern medicine, plants serve as a major source of natural products with a remarkable diversity of structural variations (David et al., 2015). Numerous novel medications have been discovered as a result of historical experiences using plants as medicinal tools. Natural products derived from plants have long been recognized for their diverse pharmacological activities. The intricate chemical structures found in natural products often contribute to their potent biological activities, making them promising candidates for therapeutic interventions (Mishra et al., 2016). The search for effective analgesics, antipyretics, and anti-inflammatory agents is a persistent challenge in medicine, driven by the need to alleviate pain, reduce fever, and mitigate inflammatory responses effectively. Natural products offer a compelling avenue in this regard as they often exhibit complex chemical profiles that interact with biological targets in unique ways. Advancements in analytical methods have significantly enhanced the isolation and characterization of bioactive compounds from plants, allowing researchers to identify specific molecules with analgesic, antipyretic, and anti-inflammatory activities (Porras et al., 2020).

Compounds isolated from plants play crucial roles in the development of treatments for various diseases. These compounds often act through diverse mechanisms, including inhibition of cyclooxygenase (COX) enzymes and modulation of inflammatory mediators such as prostaglandins, to mitigate inflammation (Süntar, 2020). Moreover, the global interest in sustainable and natural alternatives to synthetic drugs has amplified the importance of exploring natural products for pharmacological activities. Plants represent a renewable resource with immense chemical diversity, offering a promising avenue for discovering new analgesics, antipyretics, and anti-inflammatory agents that are effective yet potentially safer and more sustainable than their synthetic counterparts (Veeresham, 2012).

*Hypericum*, with 50 genera and 1,200 species, is a large genus of herbs and shrubs found widely in temperate climates worldwide. In Pakistan, nine species of this genus are known and are considered a rich source of flavones, lactones, and xanthones (Ravisankar et al., 2016). *Hypericum oblongifolium Wall* (Hypericaceae), locally known as “Pinli,” is an evergreen, widespread shrub in Himalayas and China, growing at elevations of five to six thousand feet. It is a flowering plant 6–12 m in height. Scientific interest in *H. oblongifolium* extends to its phytochemical composition, with researchers isolating bioactive compounds for their potential pharmaceutical applications, particularly in pain management and inflammation (V Simoben et al., 2015). The extract of this plant has traditionally been used to treat various ailments. Their antidepressant and antinociceptive effects have also been studied. According to the literature, the crude extract of this plant possesses potent analgesic, antipyretic, anti-inflammatory, anti-glycation, antioxidant, and anti-lipid peroxidation activities. Additionally, it shows relatively potent anti-proliferative activities (Ali et al., 2014). To date, several phytochemicals have been isolated from this plant, such as folecitin and folenolide. However, their biological potential remains unclear (Farooq et al., 2022).

**FIGURE 1:**
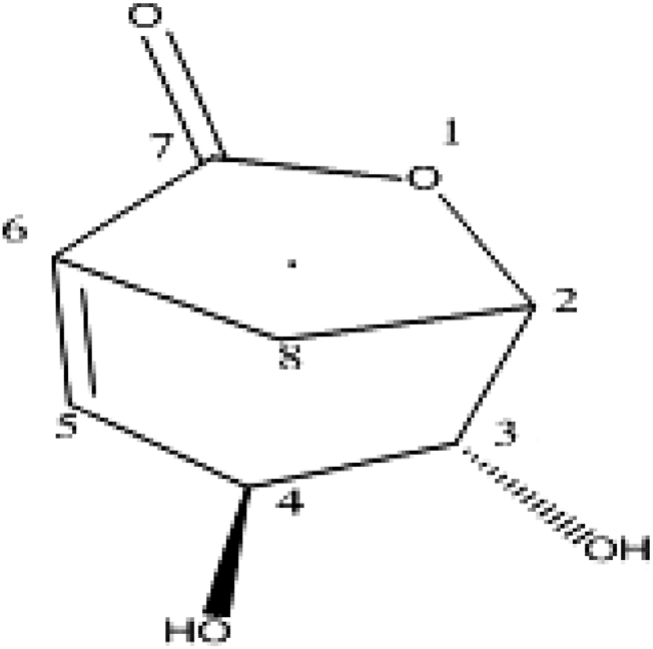
Chemical structure of folenolide.

Folenolide (3, 4-dihydroxy-6-oxabicyclo [3.2.1] oct-1-en-7-one) (Figure 1) is a lactone isolated from *H. oblongifolium.* It occurs as white crystals with a molecular formula of C_7_H_8_O_4_. In the present study, folenolide was investigated for the first time because of its pharmacological and molecular docking properties, revealing its potential as a lead compound in the development of novel analgesic, anti-inflammatory, and antipyretic agents.

## 2 Methods

### 2.1 Animal model

The pharmacological potential of folenolide was determined using adult BALB/c mice (weighing 18–25 g), which were housed under standard laboratory conditions (12 h light/dark cycle, 22 °C ± 2 °C). The animals, with an equal distribution of male and female mice, were allocated into groups (n = 6). All procedures were performed with a commitment to minimize animal pain and distress. Ethical approval was obtained under reference number 203/EC-FLES-UOP/2023 from the Institutional Ethical Review Board of the Department of Pharmacy, University of Peshawar, in accordance with the Animals (Scientific Procedures) Act, 1986.

### 2.2 Acute toxicity studies

The acute toxicities of the test compounds were also investigated according to OECD guidelines. Female BALB/c mice (n = 6 per group) were randomly divided into three groups, and the test compound (folenolide) was administered via an intraperitoneal route at doses of 25, 50, and 100 mg/kg. Following treatment, each animal was monitored closely at least once within the first 30 min and on a frequent basis over the following 24 h, with special attention paid to the first 4 h. The observations were recorded for varied tremor, convulsions, salivation, diarrhea, lethargy, and death manifestations (Raziq et al., 2015).

### 2.3 Analgesic activity

#### 2.3.1 Acetic acid-induced writhing test

Folenolide was administered to experimental animals in accordance with a previously described protocol. After treatment with test doses of 3 and 6 mg/kg, the animals were intraperitoneally injected with 1% acetic acid. The number of writhes produced by abdominal contraction, trunk twisting, extension, and elongation of the body and limbs was counted (Farooq et al., 2022). The analgesic activity was calculated using the following formula:

$$\%\text{ analgesic effect}=100-\frac{\text{No}.\;of\;writhes\;in\;tested\;animals}{\text{No}.\;\text{of}\;\text{writhes}\;\text{in}\;\text{control}\;animals}\times 100.$$

#### 2.3.2 Hot-plate analgesic test

The hot-plate test was used to determine the central analgesic potential of the test compound (19). BALB/c mice of either sex, with 18–22 g body weight, were used and provided free access to a laboratory diet and water*.* Initially, the animals were subjected to a hot-plate apparatus to record the latency time at 52 ± 0.1 ℃ by measuring the time to hind paw licking or jumping (thermal pain) of the mice upon placement on the hot plate, and animals with latency times exceeding 15 s were rejected. The test compound was administered intraperitoneally at 3 and 6 mg/kg doses, and the latency time was determined at 0, 30, 60, 90, and 120 min and compared with the saline group using diclofenac sodium at 10 mg/kg as the standard (Sajid et al., 2022). To prevent tissue damage, 30 s was imposed as the cut-off time. The percentage of analgesia was determined as follows:

$$\%\text{ Analgesia}=\frac{Test\;latency-control\;latency}{\text{Cut}\;off\;time-control\;latency}\times 100.$$

#### 2.3.3 Formalin-induced analgesic activity

This behavioral test was used to assess pain in the animal models in two phases: the acute neurogenic phase (first 5 min) and the late inflammatory phase (after 20 min). Different animal groups received either the test compound (3 or 6 mg/kg) or paracetamol, followed by an injection of formalin (2.5%, 20 μL) in the hind paw after a 1 h delay. The animals’ responses were observed by recording the licking and biting times of the affected paw (Dai et al., 2021).

### 2.4 Anti-inflammatory activity

#### 2.4.1 Carrageenan-, formalin-, and histamine-induced anti-inflammatory activity

Suppression of paw edema by the test compound was evaluated in carrageenan-, formalin-, and histamine-induced inflammatory mouse models. The test compound was administered intraperitoneally at doses of 3 and 6 mg/kg, followed by subcutaneous injection of 0.1 mL of 1% carrageenan, 20 μL of 2.5% formalin, and 0.1 mL of histamine (1 mg/mL) in normal saline after 30 min for the induction of edema (Dai et al., 2021). Paw volumes were measured using a plethysmometer, and percent inhibition of edema by the test compound in all three stimuli was measured using the following equation:

$$\text{Relative paw edema}=\frac{V2-V1}{V1}\times 100,$$

where V1 and V2 are paw volumes before formalin injection and after drug and formalin injection, respectively.

#### 2.4.2 Xylene-induced ear edema model

In the xylene-based model, mice were divided into four groups, and 2 mg/kg indomethacin (standard) and 3 and 6 mg/kg test compounds were administered orally to each group. After 1 h of sample administration, the right ear was smeared with xylene (50 μL) using the left ear as a control. All animals were sacrificed after 1 h of smearing, and both ears were severed from their bodies and weighed. The degree of ear edema was calculated as the percentage difference between the right and left ears (Labib et al., 2018).

### 2.5 Antipyretic activity

The antipyretic effects of the test compounds were investigated in BALB/c mice at doses of 3 mg/kg and 6 mg/kg. After the initial body temperature reading, a dose of 10 mL/kg of 20% Brewer’s yeast was administered subcutaneously to each mouse. After recording the temperature, the test and standard drugs were administered again, and the temperature was recorded at periodic intervals of 1, 2, 3, 4, and 5 h using a rectal thermometer probe and compared with the control group readings (Ulker et al., 2020).

### 2.6 Docking studies

Molecular docking studies were performed using AutoDock Vina (version 1.1.2) to investigate the potential binding sites of folenolide. The three-dimensional structures of the target proteins lipoxygenase (1IK3), COX-II (5KIR), tankyrase (3UA9), histamine receptor (3RZE), and opioid receptor (5C1M) were obtained from the Protein Data Bank and prepared by removing water molecules and adding polar hydrogen atoms. The folenolide structure was sketched, and energy was minimized using the MMFF94 force field to ensure a stable, low-energy conformation for docking. The docking grid was centered on the active site of each protein. The dimensions of the box were set to 20 × 20 × 20 Å for lipoxygenase, histamine, and opioid receptors and to a slightly larger 22 × 22 × 22 Å for COX-II and tankyrase to fully encompass their binding pockets. The docking search parameter “exhaustiveness” was set to ensure a comprehensive scan, and potential binding poses for each simulation were generated. To validate the accuracy of the docking protocol, the ligand was re-docked. The original ligand co-crystallized with each protein was extracted and then re-docked into its native binding site. The resulting pose was compared to the original crystal structure pose by calculating the root-mean-square deviation (RMSD). For all targets, RMSD between the docked pose and the original crystal structure pose was less than 2.0 Å, confirming the reliability of the method for reproducing known binding modes. All binding affinity values are reported in kcal/mol (Budylin et al., 2022).

### 2.7 Statistical analysis

Data are presented as the mean ± standard error of the mean (SEM). The statistical analysis was performed using GraphPad Prism software (version 5.01, GraphPad Software Inc., San Diego, CA, United States). For comparisons across multiple groups at single time points (e.g., percent inhibition in the writhing test and edema volume), a one-way analysis of variance (ANOVA) was performed. This was followed by Dunnett’s *post hoc* test to compare each treatment group (folenolide 3 and 6 mg/kg and standard drug) with the control group. For data involving repeated measurements over time, e.g., paw volume in the carrageenan-induced edema model and antipyretic activity, a two-way ANOVA was used, with time and treatment as the two factors, followed by a *post hoc* test to compare the treatment groups with the control group at each specific time point. A *p*-value (*p* < 0.05) was considered statistically significant.

## 3 Results

### 3.1 Acute toxicity

The acute toxicity studies were conducted according to the Organization for Economic Cooperation and Development (OECD) guidelines. During the 2-day observational period, no gross detrimental effects, behavioral changes, or mortality were observed after a single administration.

### 3.2 Analgesic activity

#### 3.2.1 Acetic acid-induced peripheral pain

**FIGURE 2:**
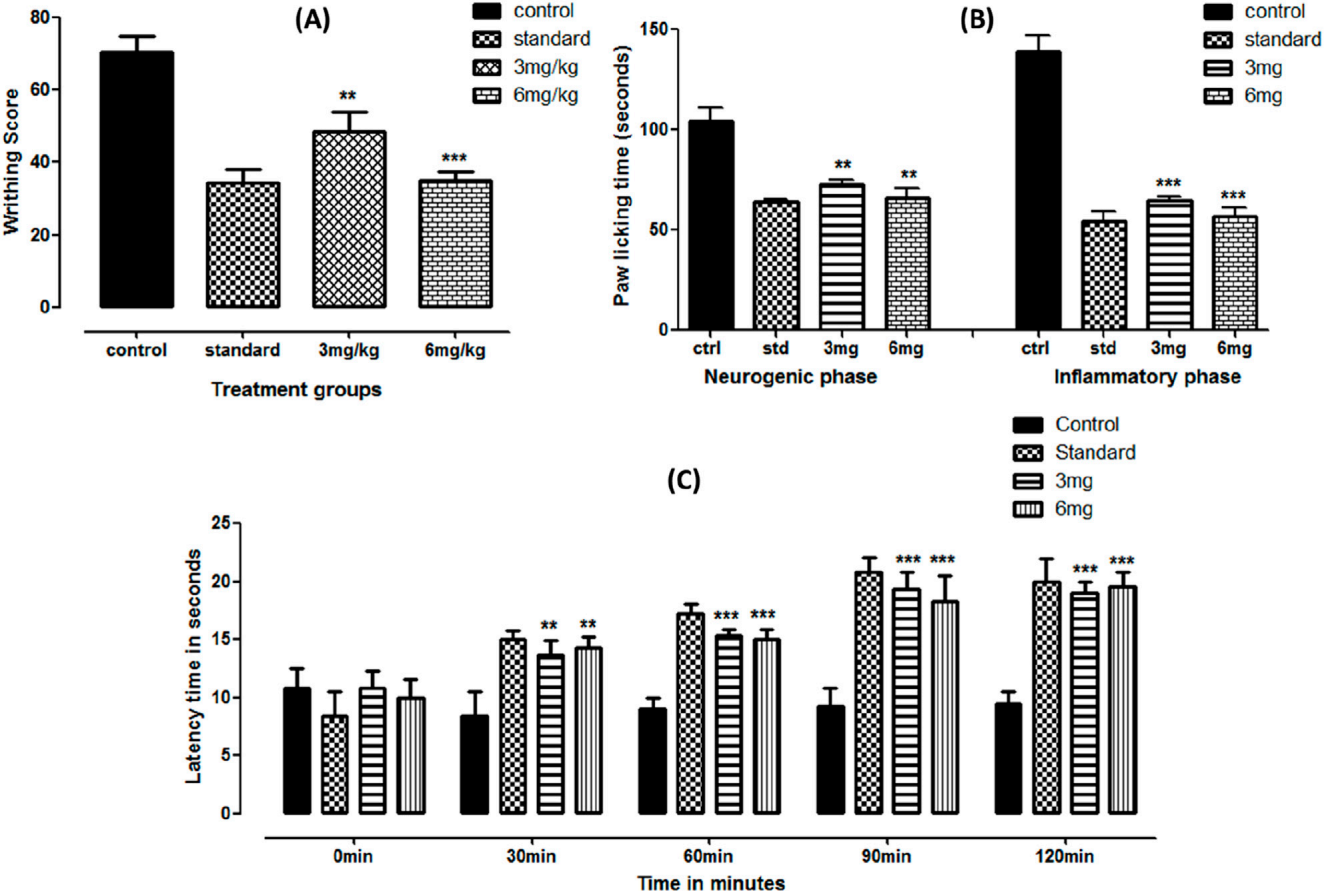
Acetic acid- **(A)**, formalin- **(B)**, and hot-plate **(C)**-based analgesic activities of folenolide. Data were analyzed by one-way ANOVA followed by Dunnett’s post hoc test. The significance levels with *p*-values < 0.5, 0.1, and 0.01 are presented as *, **, and ***, respectively.

The peripheral analgesic potential of the test compound was observed using the acetic acid-induced pain model with mefenamic acid as the standard. Significant results were observed in the given treatment doses. Approximately 31.07% and 50.13% inhibition were recorded at 3 and 6 mg doses, respectively. The data are presented in Figure 2A.

#### 3.2.2 Formalin-induced analgesic activity

Formalin-induced anti-inflammatory activity was based on two phases: the early neurogenic phase (activating nerves) and the inflammatory phase (activating inflammatory mediators). Significant effects were observed at early and late phases (*p* < 0.05). The paw-licking time was profoundly reduced at both doses, equivalent to the standard reading. While comparing the dose-dependent response in both phases, statistically insignificant (*p* > 0.05) suppression was observed between applied doses, as shown in Figure 2B.

#### 3.2.3 Hot-plate-based central analgesia

Central analgesia was observed using the hot-plate latency time test. Compared to the control, the test compound showed significantly enhanced latency time (*p* < 0.0001) at both doses, comparable with standard treatment, as shown in Figure 2C. An almost two-fold increase was observed compared to the control at 90 and 120 min of treatment.

### 3.3 Anti-inflammatory activity

#### 3.3.1 Carrageenan-induced anti-inflammatory effects

**FIGURE 3:**
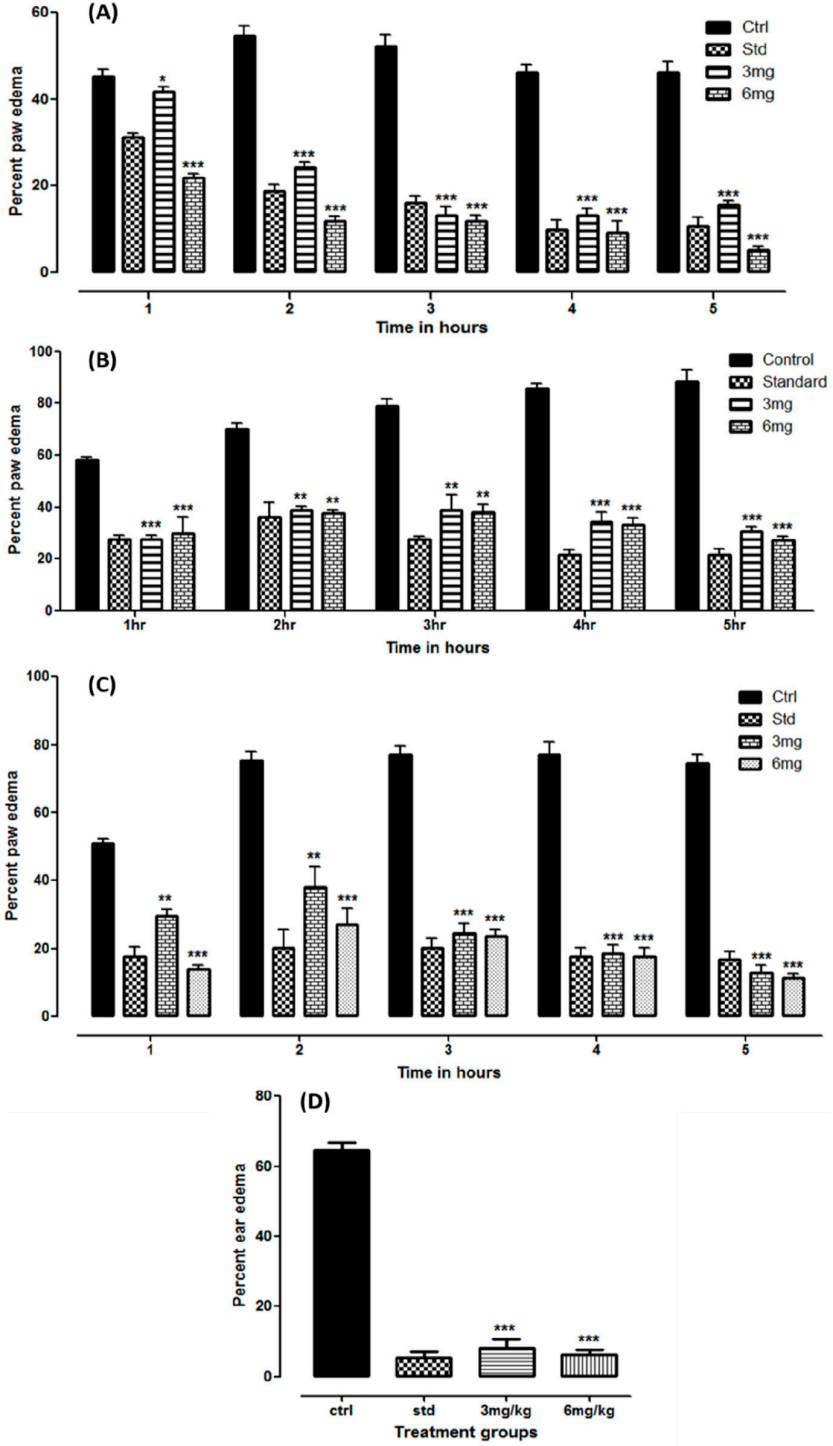
Anti-inflammatory studies on folenolide against carrageenan- **(A)**, formalin- **(B)**, histamine- **(C)**, and xylene **(D)**-induced edema in mice. Data were analyzed by one-way ANOVA followed by Dunnett’s post hoc test. The p-values < 0.5, 0.1, and 0.01 are presented as *, **, and ***, respectively.

A study on carrageenan-induced anti-inflammatory revealed a significant reduction (*p* < 0.05) in paw edema at both administered doses. The results were compared with the standard treatment. With reference to the baseline readings, the percent suppression observed in the standard, 3, and 6 mg dose groups was 89.3%, 84.3%, and 94.98%, respectively, at the fifth hour of treatment. The data are presented as the mean volume (mL) ± SD, with the *p*-value shown in Figure 3A.

#### 3.3.2 Formalin-based paw edema

The test compound was also tested against formalin-induced paw edema in a mouse model. The readings were observed for 5 h after administration. The results observed were significant (*p* < 0.05) at both doses and standard compared to the control group. Compared with the baseline readings, the overall suppression of edema in the standard, 3, and 6 mg dose groups was 78.26%, 69.17%, and 72.88%, respectively. The data are depicted as volume in ml ±SD, with the *p*-value shown in Figure 3B.

#### 3.3.3 Anti-histamine-induced anti-inflammatory effects

Similarly, significant suppression of inflammation was observed in histamine-induced paw edema at both administered dose levels (*p* < 0.05). The results were even more prominent than those in the standard group. With reference to the baseline readings in each group, total suppression of edema observed in the standard, 3, and 6 mg dose groups after 5 h was 83.12%, 87.29%, and 88.6%, respectively. The data are tabulated as the mean ± SD, with the *p*-value shown in Figure 3C.

#### 3.3.4 Xylene-induced ear edema model

The results of the xylene-induced anti-inflammatory study conducted on mouse ears were expressed as the mean ± SD and in percentage, as shown in Figure 3D. A significant reduction in weight gain was observed in the treatment groups compared with that in the control (*p* < 0.05). The weight gain observed at 3 and 6 mg doses was 8.19% ± 2.35% and 6.19% ± 1.57%, respectively, comparable to that of the standard treatment (5.31% ± 1.63%).

### 3.4 Antipyretic activity using brewer’s yeast

The antipyretic activity of the test compound was evaluated on brewer’s yeast-induced pyrexia in a mouse model. The temperature was recorded for 5 h after drug administration, as shown in Table 2. A significant reduction in body temperature was observed at both 3 and 6 mg doses (*p* < 0.0001) comparable to that of the standard treatment. The data are tabulated as the mean ± SD after recording the temperature at each interval.

### 3.5 Molecular docking studies

The docking results indicated that folenolide interacts with the selected targets with binding energies ranging from −4.4 to −5.9 kcal/mol (Table 3). Significant interactions were observed with lipoxygenase (1IK3, ΔG = −5.9 kcal/mol), and optimal binding was noted for other targets, including COX-II (5KIR), tankyrase (3UA9), histamine receptor (3RZE), and opioid receptor (5C1M), with binding energies ranging from −4.4 to −5.7 kcal/mol. Although these values are moderate compared to those of some high-affinity synthetic drugs, they are well within a biologically meaningful range, particularly for a natural product with a low molecular mass (Overington et al., 2006; Hajduk and Greer, 2007). The significant analgesic, anti-inflammatory, and antipyretic effects observed *in vivo* suggest that this moderate affinity across multiple targets may be the basis for a polypharmacological mechanism of action. Instead of potently blocking a single pathway, folenolide could exert its overall effect through the combined, synergistic modulation of several key targets involved in pain and inflammation, such as COX-II, lipoxygenase, and the histamine receptor (Anighoro et al., 2014). This multi-target profile is a common and valuable characteristic of many effective natural therapeutics, in which the collective effect of several moderate interactions can result in a potent pharmacological outcome with a potentially improved safety profile (Atanasov et al., 2021).

## 4 Discussion

The assessment of acute toxicity is essential in evaluating the safety profile of pharmaceutical agents (Mondal et al., 2019). The results of the study (Table 1) revealed the safety of folenolide as no mortality was recorded. This indicates that folenolide exhibits promising safety characteristics in acute toxicity assessments.

**TABLE 1:** Acute toxicity of folenolide.

Dose (mg/kg)	Gross effect after 4 h	Mortality after 24 h
25	Alive and normal	No mortality observed
50	Alive and normal	No mortality observed
100	Alive and normal	No mortality observed

Folenolide demonstrated strong analgesic properties in both peripheral and central pain models. In the acetic acid-induced writhing assay, folenolide at 3 and 6 mg/kg resulted in dose-dependent inhibition of writhing by 31.07% and 50.13%, respectively, indicating a peripheral analgesic effect comparable to that of mefenamic acid, as shown in Figure 2A. The efficacy of folenolide demonstrated enhanced potency compared to other well-known phytochemicals, including curcumin, as a dose of 50 mg/kg of it is required to produce similar analgesic effects (Mohammed and Sabry, 2020). These findings suggest its promising peripheral analgesic activity, like other bioactive compounds (Singsai et al., 2020). Similarly, significant reductions in paw-licking time were observed in both phases of the formalin test, indicating potential analgesic and anti-inflammatory effects of folenolide, as shown in Figure 2B. Formalin-induced pain is characterized by two distinct phases: the early neurogenic phase, involving direct activation of pain-sensing nerves, and the late inflammatory phase, characterized by the release of inflammatory mediators (Turnaturi et al., 2023). Finally, in the hot-plate test, folenolide significantly prolonged latency times at 90 and 120 min post-administration compared to the control, highlighting its sustained central analgesic activity, as shown in Figure 2C. These results align with the analgesic actions commonly observed in natural compounds, which frequently act by modulating central and peripheral system pain pathways (Liktor-Busa et al., 2021; Jabbari et al., 2024).

The test compound demonstrated strong anti-inflammatory effects across various established *in vivo* models (Matos et al., 2021; Oqal et al., 2023). Figure 3A demonstrates that significant reductions (*p* < 0.05) in paw edema were observed in both dose groups, where the group that was administered the 6 mg dose showed the highest suppression (94.98%) at the fifth hour interval, comparable to the standard treatment (89.3%) in the carrageenan-induced anti-inflammatory study. This showed the potent anti-inflammatory effects of folenolide compared to other bioactive natural compounds, including quercetin, which often requires doses above 25 mg/kg to achieve similar effects (Batiha et al., 2020). Formalin-induced paw edema serves as a critical tool in preclinical research (Horváth et al., 2021). The efficacy of folenolide against formalin-induced paw edema in a mouse model revealed significant anti-inflammatory effects. Over the 5-h observation period, the study showed reductions in paw edema volume, with percent suppressions of 78.26%, 69.17%, and 72.88% for the standard, 3, and 6 mg dose groups, respectively, compared to baseline measurements. These findings (Figure 3B) indicate the potential of folenolide to modulate inflammatory mediators involved in edema formation (Ferraz et al., 2020). Figure 3C shows that folenolide antagonized the inflammation induced by histamine. Significant reductions (*p* < 0.05) in paw edema were observed at both dose levels. However, animals that received a higher dose (6 mg) showed the highest suppression (88.6%) after 5 h, surpassing the standard treatment (83.12%). Folenolide may alleviate histamine-induced inflammation pathways (Almasarwah et al., 2023). In the xylene-induced ear edema model, the anti-inflammatory effect is calculated as the percentage weight gain of the ear. The low values of 6.19%–8.19% observed in the treatment groups indicate a very strong suppression of edema (approximately 92%–94% inhibition compared to the control), which is consistent with the high levels of efficacy observed in the paw edema models. Xylene induces localized inflammation, making it a suitable model for evaluating anti-inflammatory agents (Basit et al., 2022; Rousdy et al., 2022). The results from Figure 3D suggest a dose-dependent anti-inflammatory response of the test compound.

Brewer’s yeast is a useful test for the screening of isolated compounds for their antipyretic effect (Mohankumar et al., 2022; Negi et al., 2023). Yeast-induced pyrexia could be the production of prostaglandins, and it can be achieved by blocking the cyclo-oxygenase enzyme activity (Chansiw et al., 2024; Abd-Elhak et al., 2024; Veena et al., 2022). Folenolide significantly attenuated the rectal temperature of mice during the 5-hour observation period, and consistently lowered rectal temperatures indicate its potent antipyretic properties. In particular, the 6 mg/kg dose group showed pronounced temperature reductions from the second hour onward, achieving statistically significant differences compared to controls (Table 2). These results suggest that the test compound showed effective antipyretic activities by mediating the mechanisms that regulate fever response pathways (Ogbonna et al., 2025).

**TABLE 2:** Antipyretic activity of folenolide using brewer’s yeast.

Group	Temperature before yeast administration Mean ± SD °F	Temperature control pre-drug administration Mean ± SD °F	Rectal temperature after drug administration in mean ± SD °F (*p*-value)				
			1 h	2 h	3 h	4 h	5 h
Control	98.07 ± 0.06	101.42 ± 0.38	101.65 ± 0.41	101.68 ± 0.18	101.80 ± 0.16	101.65 ± 0.22	101.36 ± 0.94
Standard	98.80 ± 0.50	101.59 ± 0.21	99.30 ± 0.52 (0.0035)**	99.13 ± 0.51 (0.0012)**	98.83 ± 0.68 (0.0018)**	98.90 ± 0.45 (0.0006)***	98.53 ± 0.21 (0.0001)***
3 mg/kg	98.80 ± 0.36	101.53 ± 0.37	98.57 ± 0.26 (0.0004)***	98.43 ± 0.23 (<0.0001)***	98.50 ± 0.30 (<0.0001)***	98.43 ± 0.17 (<0.0001)***	98.47 ± 0.30 (0.0002)***
6 mg kg	98.50 ± 0.15	101.09 ± 0.24	99.30 ± 0.62 (0.0054)**	99.20 ± 0.72 (0.0044)**	99.20 ± 0.20 (<0.0001)***	98.60 ± 0.09 (<0.0001)***	98.63 ± 0.11 (<0.0001)***

**FIGURE 4:**
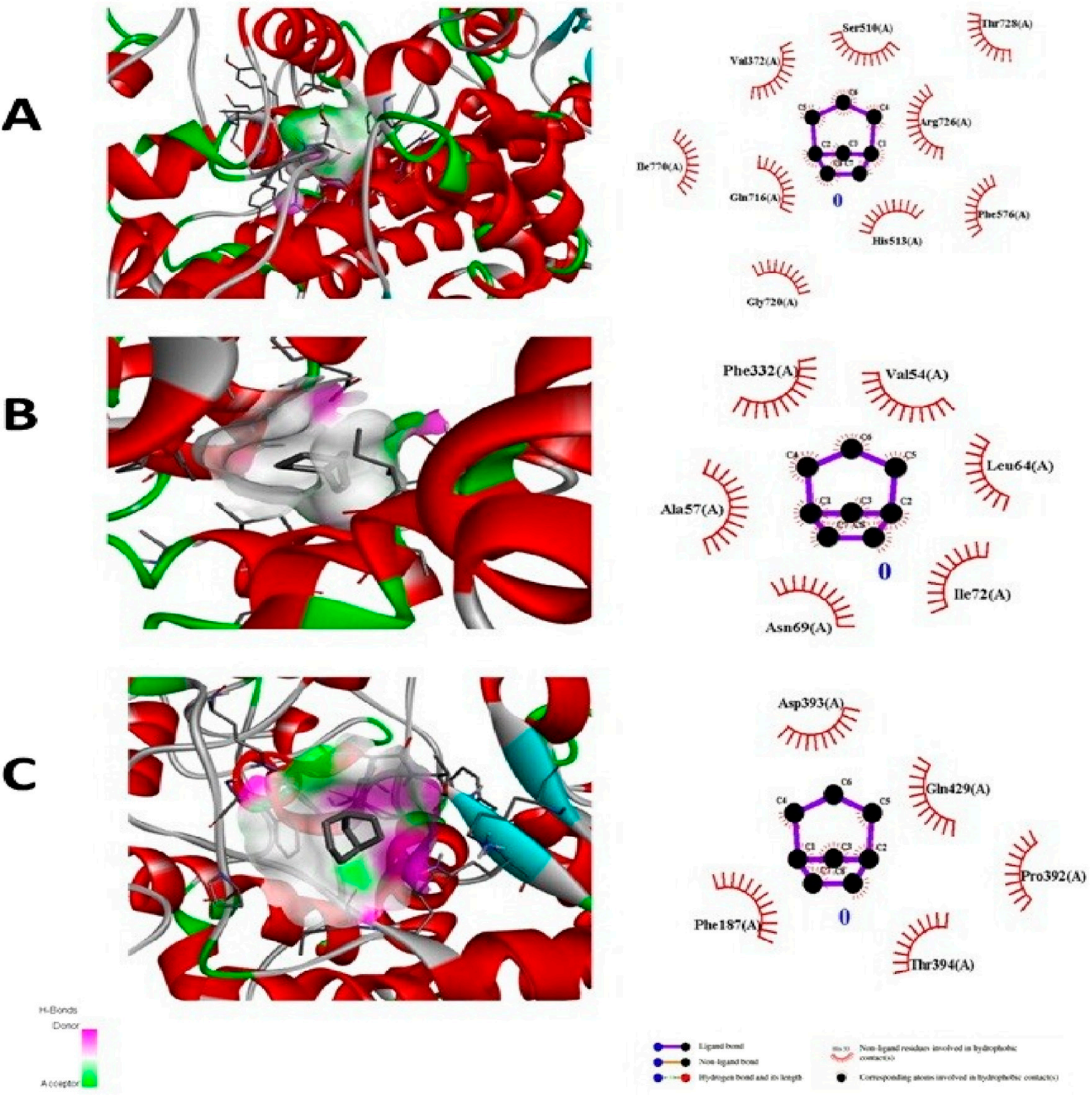
Molecular docking analysis of folenolide illustrating binding poses and interactions with **(A)** 1IK3, **(B)** 2R4R, and **(C)** 5K1R. Interactions are mediated by van der Waals, π–π, π–sigma, and π–alkyl forces.

**FIGURE 5:**
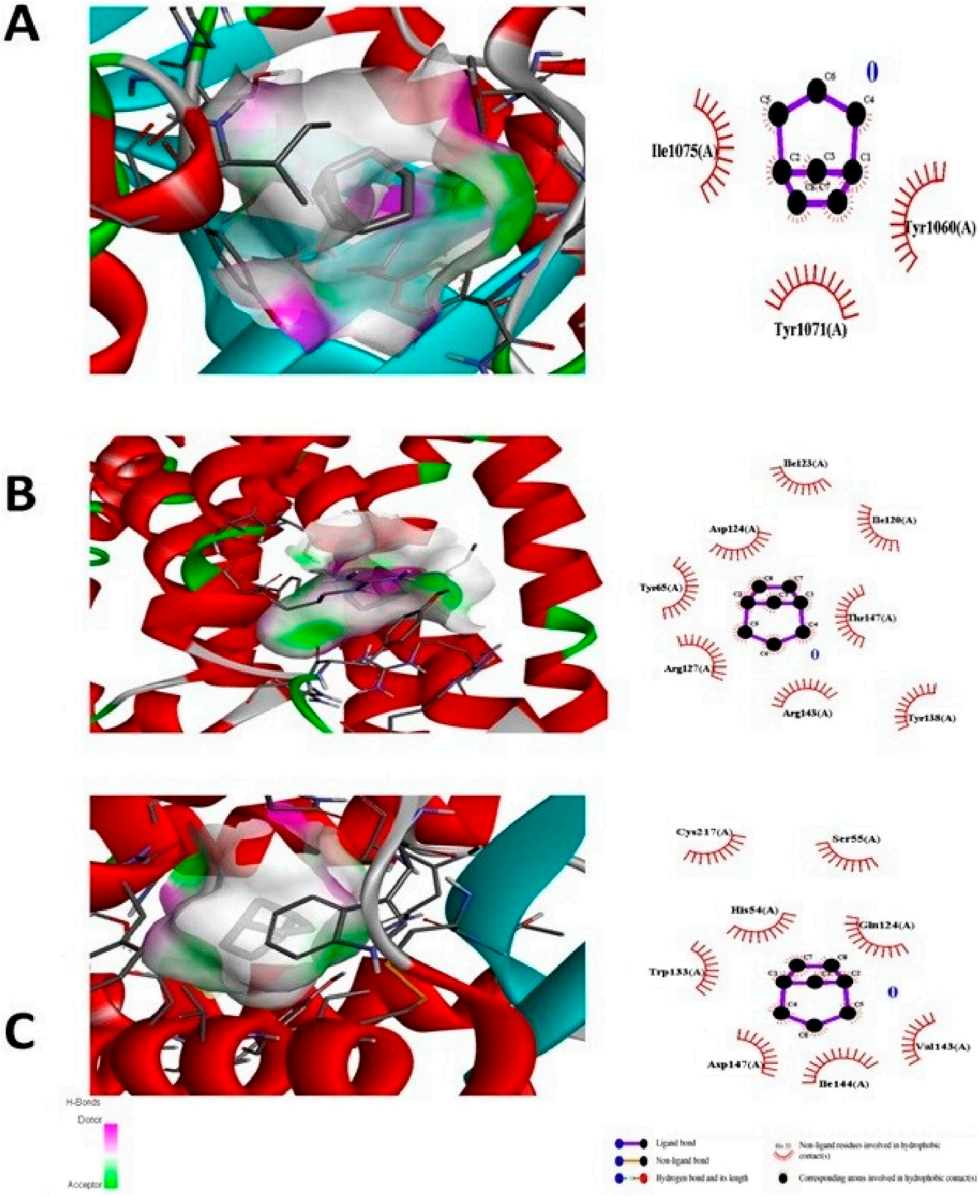
Molecular docking analysis of folenolide illustrating binding poses and interactions with **(A)** 3UA9, **(B)** 3RZE, and **(C)** 5C1M. Interactions are mediated by van der Waals, π–sigma, and π–alkyl forces.

The test compound was docked against transcription regulators 1IK3 (15 lipoxygenase), 2R4R, 5KIR [COX (cyclooxygenase-2, COX-2)], 3UA9 (tankyrase-1 and tankyrase-2), 5C1M (opioid receptors), 3RZE (histaminic receptors), and 1IK3 (Tegegne and Alehegn, 2023). The highest free binding energy (ΔG) was observed with pose 1 (−5.9 ΔG (kcal/mol). More importantly, N H-bonding interaction was observed with any pose (1–9) that shows less affinity for binding interactions. Furthermore, hydrophobic interactions of folenolide were noticed with Gly720, Gln716, Ille770, Val372, Ser510, Thr728, Arg726, Phe576, and His513, which were mainly due to van der Waals forces and π–alkyl interactions, reflecting weak interactions with the target gene *1IK3*. In the case of the transcription regulator 2R4R, no H-binding was observed in docking analysis. However, the highest free energy (ΔG) was recorded in the case of pose 1 [−5.4 ΔG (kcal/mol)]. In interaction analysis, weak interactions of the ligand were recorded with Ala57, Phe332, Val54, Leu64, Ile72, and Asn69 amino acid residues, which were due to van der Waals forces and π–alkyl interactions (Table 3; Figure 4). Nevertheless, the transcriptional regulator 5KIR did not participate in H-bonding interactions with the tested ligand (poses 1–9). In this case, the highest free binding energy (ΔG) was observed in the case of pose 1 (−4.4 ΔG kcal/mol), which was again less than that of other targets. The neighboring interacting amino acids included Phe187, Asp393, Gln429, Pro392, and Thr394. The π–sigma, van der Waals forces, and π–alkyl interactions contributed to these binding interactions (Table 3). In the molecular docking of the ligand molecule with the target gene *3UA9*, only weak interactions were recorded with Asp147, Trp133, His54, Cys217, Ser55, Gln214, Val143, and Ile144 amino acids, with the highest binding free energy (ΔG) (−5.7ΔG (kcal/mol)) with pose 1 compared to that of all other poses (up to 9). In this case, no H-binding interactions were noticed (Table 3; Figure 4). In the docking of the ligand with 3RZE, no H-bond formation was observed, indicating no interaction with the target. Furthermore, weak interactions were recorded with Arg127, Tyr65, Asp124, Ile123, Thr147, Tyr138, and Arg143, with the highest free energy [−5.3 ΔG (kcal/mol)], which was due to π–sigma, van der Waals forces, and π–alkyl interactions (Table 3; Figure 5). Finally, no H-bond formation was recorded with 5C1M. However, weak interactions were recorded with Asp147, Trp133, His54, Cys217, Ser55, Gln124, Val143, and Ile144. In this case, interacting forces included weak alkyl and van der Waals forces, with the highest free energy (ΔG) [−5.0 ΔG (kcal/mol)] in pose 1 (Table 3; Figure 5). Weak intermolecular interactions, such as hydrogen bonding and hydrophobic interactions, are key players in stabilizing energetically favored ligands within the open conformational environment of protein structures (Beatrice, 2022). Weak intermolecular interactions, such as H-bonding and hydrophobic (π–π, π–amide, π–cation/anion, π–alkyl, and π–σ) interactions, are of great significance since they determine the stability and structure of proteins that are crucial for therapeutic effects (Siddique et al., 2022; Rafey et al., 2022). Hydrogen-bonding interaction governs the stability of the host–guest complex. However, the presence of multiple hydrophobic interactions competes with hydrogen bonding interaction in several conformers, resulting in a decrease in binding energy (Deivasigamani et al., 2024). In current investigations, the interactions were mainly ruled by hydrophobic forces and, therefore, play a key role in the therapeutic efficacy of folenolide. Furthermore, hydrophobic interactions play an important role in the folding of proteins, contributing to the stability and biological activity of the protein moiety (Berishvili et al., 2020). This allows the protein to reduce the surface area and decrease the unwanted interactions with water (Huang et al., 2015). It is thus concluded that folenolide has strong interactions with the tested molecule and the ability to interact with key targets involved in analgesia and inflammation, indicating that the potent *in vivo* effects observed may, therefore, be attributed to a polypharmacological mode of action.

**TABLE 3:** Docking score, with H- and non-H-bonding interactions against different targets.

Binding free energy ΔG (kcal/mol)	Pose no.	H-bond	H-bond interaction residue	Neighbor interacting residue
1IK3				
−5.9	1	0	0	Gly720, Gln716, Ille770, Val372, Ser510, Thr728, Arg726, Phe576, and His513
2R4R				
−5.4	1	0	0	Ala57, Phe332, Val54, Leu64, Ile72, and Asn69
5KIR				
−4.4	1	0	0	Phe187, Asp393, Gln429, Pro392, and Thr394
3UA9				
−5.7	1	0	0	Asp147, Trp133, His54, Cys217, Ser55, Gln214, Val143, and Ile144
3RZE				
−5.3	1	0	0	Arg127, Tyr65, Asp124, Ile123, Thr147, Tyr138, and Arg143
5C1M				
−5.0	1	0	0	Asp147, Trp133, His54, Cys217, Ser55, Gln124, Val143, and Ile144

## 5 Conclusion

Pharmacological evaluation and molecular docking studies have demonstrated the significant potential of folenolide as a drug. It showed marked analgesic effects through both peripheral and central mechanisms, suggesting its efficacy in modulating the pain pathways. It also exhibits significant anti-inflammatory properties in various models, indicating its ability to attenuate inflammatory responses through different pathways. Moreover, its antipyretic activity indicated its potential for fever management. Molecular docking studies have suggested that folenolide exerts its effects by modulating protein structures involved in the pain, inflammation, and fever pathways. In conclusion, the findings from this study collectively support the promising pharmacological profile of folenolide and its potential as a natural-product-based therapeutic option for pain and inflammatory disorders.

## Data Availability

The original contributions presented in the study are included in the article/supplementary material; further inquiries can be directed to the corresponding authors.

## References

[B1] Abd-Elhak N. Sayed H. Hashem M. (2024). Fennel, carob, rosemary extracts as AntiInflammatory activity functions on formalin induced paw edema in albino rats. Food Technol. Res. J. 3, 14–32. 10.21608/ftrj.2024.341747

[B2] Ali M. Latif A. Zaman K. Arfan M. Maitland D. Ahmad H. (2014). Anti-ulcer xanthones from the roots of Hypericum oblongifolium wall. Fitoterapia 95, 258–265. 10.1016/j.fitote.2014.03.014 24685505

[B3] Almasarwah S. Y. Oran S. A. Darwish R. M. (2023). Efficacy of Nitraria retusa L. fruits aqueous and methanol extracts as antioxidant and anti-inflammatory activities on carrageenan-induced paw edema in rats. Trop. J. Nat. Prod. Res. 7.

[B4] Anighoro A. Bajorath J. Rastelli G. (2014). Polypharmacology: challenges and opportunities in drug discovery. J. Med. Chem. 57, 7874–7887. 10.1021/jm5006463 24946140

[B5] Atanasov A. G. Zotchev S. Dirsch V. Supuran C. (2021). Natural products in drug discovery: advances and opportunities. Nat. Rev. Drug Discov. 20, 200–216. 10.1038/s41573-020-00114-z 33510482 PMC7841765

[B6] Basit A. Shutian T. Khan A. Khan S. M. Shahzad R. Khan A. (2022). Anti-inflammatory and analgesic potential of leaf extract of Justicia adhatoda L.(Acanthaceae) in carrageenan and Formalin-induced models by targeting oxidative stress. Biomed. and Pharmacother. 153, 113322. 10.1016/j.biopha.2022.113322 35763968

[B7] Batiha G. E.-S. Beshbishy A. M. Ikram M. Mulla Z. S. El-Hack M. E. A. Taha A. E. (2020). The pharmacological activity, biochemical properties, and pharmacokinetics of the major natural polyphenolic flavonoid: quercetin. Foods 9, 374. 10.3390/foods9030374 32210182 PMC7143931

[B8] Beatrice A. (2022). “Evaluation of anti-pyretic activity of the siddha poly herbomineral formulation oma chooranam in brewer’s yeast induced pyrexia,” in Wistar albino rats.

[B9] Berishvili V. P. Kuimov A. N. Voronkov A. E. Radchenko E. V. Kumar P. Choonara Y. E. (2020). Discovery of novel tankyrase inhibitors through molecular docking-based virtual screening and molecular dynamics simulation studies. Molecules 25, 3171. 10.3390/molecules25143171 32664504 PMC7397142

[B10] Budylin G. S. Davydov D. A. Zlobina N. V. Baev A. V. Artyushenko V. G. Yakimov B. P. (2022). *In vivo* sensing of cutaneous edema: a comparative study of diffuse reflectance, raman spectroscopy and multispectral imaging. J. Biophot. 15, e202100268. 10.1002/jbio.202100268 34661967

[B11] Chansiw N. Chusri P. Praman S. Hawiset T. Sukhorum W. Champakam S. (2024). Anti-inflammatory potential of a Thai traditional remedy called prabchompoothaweep in an animal model of acute and sub-acute inflammation. J. Ethnopharmacol. 319, 117380. 10.1016/j.jep.2023.117380 37925003

[B12] Dai G. Li B. Xu Y. Li Z. Mo F. Wei C. (2021). Synergistic interaction between matrine and paracetamol in the acetic acid writhing test in mice. Eur. J. Pharmacol. 895, 173869. 10.1016/j.ejphar.2021.173869 33454375

[B13] David B. Wolfender J.-L. Dias D. A. (2015). The pharmaceutical industry and natural products: historical status and new trends. Phytochem. Rev. 14, 299–315. 10.1007/s11101-014-9367-z

[B14] Deivasigamani P. Rubavathy S. E. Jayasankar N. Saravanan V. Thilagavathi R. Prakash M. (2024). Dual anti-inflammatory and anticancer activity of novel 1, 5-Diaryl pyrazole derivatives: molecular modeling, synthesis, *in vitro* activity, and dynamics study. Biomedicines 12, 788. 10.3390/biomedicines12040788 38672144 PMC11048033

[B15] Farooq U. Sahibzada M. U. K. Khan T. Ullah R. Shahid M. Khusro A. (2022). Folecitin isolated from hypericum oblongifolium exerts neuroprotection against lipopolysaccharide-induced neuronal synapse and memory dysfunction via p-AKT/Nrf-2/HO-1 signalling pathway. Evidence-based Complementary Altern. Med. 2022, 9419918. 10.1155/2022/9419918 35388307 PMC8979689

[B16] Ferraz C. R. Carvalho T. T. Manchope M. F. Artero N. A. Rasquel-Oliveira F. S. Fattori V. (2020). Therapeutic potential of flavonoids in pain and inflammation: mechanisms of action, pre-clinical and clinical data, and pharmaceutical development. Molecules 25, 762. 10.3390/molecules25030762 32050623 PMC7037709

[B17] Hajduk P. J. Greer J. (2007). A decade of fragment-based drug design: strategic advances and lessons learned. Nat. Rev. Drug Discov. 6, 211–219. 10.1038/nrd2220 17290284

[B18] Horváth Á. Payrits M. Steib A. Kántás B. Biró-Süt T. Erostyák J. (2021). Analgesic effects of lipid raft disruption by sphingomyelinase and myriocin via transient receptor potential vanilloid 1 and transient receptor potential ankyrin 1 ion channel modulation. Front. Pharmacol. 11, 593319. 10.3389/fphar.2020.593319 33584270 PMC7873636

[B19] Huang W. Manglik A. Venkatakrishnan A. Laeremans T. Feinberg E. N. Sanborn A. L. (2015). Structural insights into µ-opioid receptor activation. Nature 524, 315–321. 10.1038/nature14886 26245379 PMC4639397

[B20] Jabbari S. Zakaria Z. A. Ahmadimoghaddam D. Mohammadi S. (2024). The oral administration of lotus corniculatus L. attenuates acute and chronic pain models in male rats. J. Ethnopharmacol. 319, 117181. 10.1016/j.jep.2023.117181 37734474

[B21] Labib M. B. Sharkawi S. M. El-Daly M. (2018). Design, synthesis of novel isoindoline hybrids as COX-2 inhibitors: anti-inflammatory, analgesic activities and docking study. Bioorg. Chem. 80, 70–80. 10.1016/j.bioorg.2018.05.018 30005203

[B22] Liktor-Busa E. Keresztes A. Lavigne J. Streicher J. M. Largent-Milnes T. M. (2021). Analgesic potential of terpenes derived from Cannabis sativa. Pharmacol. Rev. 73, 1269–1297. 10.1124/pharmrev.120.000046 34663685 PMC11060501

[B23] Matos M. S. Anastácio J. D. Nunes Dos Santos C. (2021). Sesquiterpene lactones: promising natural compounds to fight inflammation. Pharmaceutics 13, 991. 10.3390/pharmaceutics13070991 34208907 PMC8309091

[B24] Mishra P. Kumar A. Nagireddy A. Mani D. N. Shukla A. K. Tiwari R. (2016). DNA barcoding: an efficient tool to overcome authentication challenges in the herbal market. Plant Biotechnol. J. 14, 8–21. 10.1111/pbi.12419 26079154 PMC11388846

[B25] Mohammed H. O. Sabry R. M. (2020). The possible role of curcumin against changes caused by paracetamol in testis of adult albino rat (histological, immunohistochemical and biochemical study). Egypt. J. Histology 43, 819–834.

[B26] Mohankumar R. Prakash S. E. L. Irfan N. Mohanraj S. Kumarappan C. (2022). Evaluation of analgesic, anti-inflammatory, and antipyretic activities of Ziziphus mauritania lam leaves in animal models. Pharmacol. Research-Modern Chin. Med. 4, 100153. 10.1016/j.prmcm.2022.100153

[B27] Mondal A. Maity T. K. Bishayee A. (2019). Analgesic and anti-inflammatory activities of quercetin-3-methoxy-4′-glucosyl-7-glucoside isolated from Indian medicinal plant Melothria heterophylla. Medicines 6, 59. 10.3390/medicines6020059 31137810 PMC6631596

[B28] Negi P. Agarwal S. Garg P. Ali A. Kulshrestha S. (2023). “ *In vivo* models of understanding inflammation.” Recent developments in anti-inflammatory therapy. Elsevier.

[B29] Ogbonna U. N. Emmanuel P. D. Chizi N. G. Ive A. S. (2025). Effect of aqueous leaf extract of Macaranga barteri on baker's yeast-induced pyrexia in wistar albino rats.

[B30] Oqal M. Qnais E. Alqudah A. Gammoh O. (2023). Analgesic effect of the flavonoid herbacetin in nociception animal models. Eur. Rev. Med. and Pharmacol. Sci. 27, 11236–11248. 10.26355/eurrev_202312_34563 38095373

[B31] Overington J. P. Al-Lazikani B. Hopkins A. L. (2006). How many drug targets are there? Nat. Rev. Drug Discov. 5, 993–996. 10.1038/nrd2199 17139284

[B32] Porras G. Chassagne F. Lyles J. T. Marquez L. Dettweiler M. Salam A. M. (2020). Ethnobotany and the role of plant natural products in antibiotic drug discovery. Chem. Rev. 121, 3495–3560. 10.1021/acs.chemrev.0c00922 33164487 PMC8183567

[B33] Rafey A. Amin A. Kamran M. Aziz M. I. Athar V. Niaz S. I. (2022). Evaluation of major constituents of medicinally important plants for anti-inflammatory, antidiabetic and AGEs inhibiting properties: *in vitro* and simulatory evidence. Molecules 27, 6715. 10.3390/molecules27196715 36235251 PMC9571302

[B34] Ravisankar N. Jerrine J. Radhakrishana M. Rajasekar T. (2016). *In vitro* cytotoxicity of methanol extracts of Hypericum wightianum and hypericum hookerianuim against 3T3L1 cell lines. Bangladesh J. Pharmacol. 11, 328–329.

[B35] Raziq N. Saeed M. Shahid M. Muhammad N. Khan H. Gul F. (2015). Pharmacological basis for the use of Hypericum oblongifolium as a medicinal plant in the management of pain, inflammation and pyrexia. BMC complementary Altern. Med. 16, 41–47. 10.1186/s12906-016-1018-z 26832937 PMC4736148

[B36] Rousdy D. W. Wardoyo E. R. P. Ifadatin S. (2022). Anti-inflammatory activity of bajakah stem (Spatholobus littoralis hassk.) ethanolic extract in carrageenan-induced paw edema mice. J. Biodjati 7, 66–74. 10.15575/biodjati.v7i1.14126

[B37] Sajid A. Afzal M. Sajid A. Manzoor Q. Sharif A. Younas S. (2022). NMR structure elucidation and molecular modeling of lipoxygenase and cholinesterase inhibiting steroids from Hypericum oblongifolium. Curr. Org. Chem. 26, 1798–1806. 10.2174/1385272827666221216111557

[B38] Siddique S. Ahmad K. R. Nawaz S. K. Ali R. Ahmad S. N. Suleman S. (2022). *In-vivo* anti-inflammatory, analgesic and anti-pyretic activities of synthetic indole derivatives in mice.

[B39] Singsai K. Charoongchit P. Chaikaew W. Boonma N. Fhanjaksai P. Chaisatan K. (2020). Antilipoxygenase and anti-inflammatory activities of Streblus asper leaf extract on xylene-induced ear edema in mice. Adv. Pharmacol. Pharm. Sci. 2020, 3176391. 10.1155/2020/3176391 33354670 PMC7737438

[B40] Süntar I. (2020). Importance of ethnopharmacological studies in drug discovery: role of medicinal plants. Phytochem. Rev. 19, 1199–1209. 10.1007/s11101-019-09629-9

[B41] Tegegne B. A. Alehegn A. A. (2023). Antipyretic potential of 80% methanol extract and solvent fractions of bersama abyssinica fresen.(Melianthaceae) leaves against yeast-induced pyrexia in mice. J. Exp. Pharmacol. 15, 81–91. 10.2147/JEP.S390825 36879895 PMC9985388

[B42] Turnaturi R. Piana S. Spoto S. Costanzo G. Reina L. Pasquinucci L. (2023). From plant to chemistry: sources of active opioid antinociceptive principles for medicinal chemistry and drug design. Molecules 28, 7089. 10.3390/molecules28207089 37894567 PMC10609244

[B43] Ulker E. Caillaud M. Patel T. White A. Rashid D. Alqasem M. (2020). C57BL/6 substrain differences in formalin-induced pain-like behavioral responses. Behav. Brain Res. 390, 112698. 10.1016/j.bbr.2020.112698 32428630 PMC7375808

[B44] Veena G. Mohanan A. Bhat S. Ramesh N. (2022). An *in vivo* study to evaluate the antipyretic activity of suryaprabha gulika in brewer’s yeast induced pyrexia in wistar albino rats. J. Ayurveda Integr. Med. Sci. 7, 76–88.

[B45] Veeresham C. (2012). Natural products derived from plants as a source of drugs. J. Adv. Pharm. Technol. Res. 3, 200–201. 10.4103/2231-4040.104709 23378939 PMC3560124

[B46] V Simoben C. Ibezim A. Ntie-Kang F. N Nwodo J. L Lifongo L. (2015). Exploring cancer therapeutics with natural products from African medicinal plants, part I: xanthones, quinones, steroids, coumarins, phenolics and other classes of compounds. Anti-Cancer Agents Med. Chem. Former. Curr. Med. Chemistry-Anti-Cancer Agents 15, 1092–1111. 10.2174/1871520615666150113110241 25584692

